# Geospatial Analysis of Inflammatory Breast Cancer and Associated Community Characteristics in the United States

**DOI:** 10.3390/ijerph14040404

**Published:** 2017-04-11

**Authors:** Lia Scott, Lee R. Mobley, Dora Il’yasova

**Affiliations:** 1 School of Public Health, Georgia State University, Atlanta, GA 30302, USA; lmobley@gsu.edu (L.R.M.); dilyasova@gsu.edu (D.I.); 2 Andrew Young School of Policy Studies, Georgia State University, Atlanta, GA 30302, USA

**Keywords:** inflammatory breast cancer, geographic analysis, geospatial analysis, epidemiology, population health, poverty, rural, United States, health disparities

## Abstract

Inflammatory breast cancer (IBC) is a rare and aggressive form of breast cancer, almost always diagnosed at late stage where mortality outcomes and morbidity burdens are known to be worse. Missed by mammography screening, IBC progresses rapidly and reaches late stage by the time of diagnosis. With an unknown etiology and poor prognosis, it is crucial to evaluate the distribution of the disease in the population as well as identify area social and economic contextual risk factors that may be contributing to the observed patterns of IBC incidence. In this study, we identified spatial clustering of county-based IBC rates among US females and examined the underlying community characteristics associated with the clusters. IBC accounted for ~1.25% of all primary breast cancers diagnoses in 2004–2012 and was defined by the Collaborative Stage (CS) Extension code 710 and 730. Global and local spatial clusters of IBC rates were identified and mapped. The Mann-Whitney U test was used to compare median differences in key contextual variables between areas with high and low spatial clusters of IBC rates. High clusters are counties and their neighbors that all exhibit above average rates, clustered together in a fashion that would be extremely unlikely to be observed by chance, and conversely for low clusters. There was statistically significant evidence of spatial clustering into high and low rate clusters. The average rate in the high rate clusters (*n* = 46) was approximately 12 times the average rate in low rate clusters (*n* = 126), and 2.2 times the national average across all counties. Significant differences were found in the medians of the underlying race, poverty, and urbanicity variables when comparing the low cluster counties with the high cluster counties (*p* < 0.05). Cluster analysis confirms that IBC rates differ geographically and may be influenced by social and economic environmental factors. Particular attention may need to be paid to race, urbanicity and poverty when considering risk factors for IBC and when developing interventions and alternative prevention strategies.

## 1. Introduction

Inflammatory breast cancer (IBC) is a rare form of breast cancer with distinct clinicopathologic features characterized by rapid onset and aggressive behavior, with a median overall survival of less than four years with multimodality treatment [1,2]. Recent studies estimate that IBC accounts for between 1% and 6% of all breast cancer cases [2,3,4]. The American Joint Committee on Cancer defines IBC as a clinicopathologic entity characterized by diffuse erythema and edema, often without an underlying palpable mass [1]. This definition relies on clinical features, while considering pathologic features to support diagnosis. Given the inherent difficulty in diagnosis for IBC, there is an expectation that reported incidence trends will vary within the literature.

While it only accounts for a small percentage of all breast cancers, disparities in IBC rates exist. Reports have found that IBC cases in the United States occurred at significantly higher rates in black women as compared to white women, and at younger ages [1,2,5]. Once diagnosed, survival differs by race as well. Schlichting et al. found that black women had a significantly worse probability of survival from IBC compared to white women, regardless of socioeconomic environment [6].

Besides race, socioeconomic status has been linked to the diagnosis of IBC [6,7,8]. Additionally, Schlichting et al. found survival to be worse for those residing in a lower socioeconomic status or in non-metro counties, and that survival was worse for black women regardless of inflammatory status and socioeconomic status, in unadjusted models. However, survival differences were not found when they adjusted for age at diagnosis, race, tumor and treatment characteristics [6]. No studies have found living in a rural area to be a significant predictor of IBC, however rural areas are often confounded by poverty and low educational attainment and lower access to and utilization of healthcare—which have been significant predictors [9].

Given the aggressive nature of the disease and late diagnosis, it is crucial to determine risk factors associated with IBC. This study aims to fill gaps in the literature through the use of geospatial analysis. Due to the rarity of IBC, it is important to identify potential ”hot-spots” where disease occurrence may be higher than average, in order to better isolate potential risk factors for the disease. If the social and physical contexts of these “hot-spots” are similar, we are better able to identify risk factors and develop intervention and prevention strategies. Geospatial analysis allows us to explore these relationships within the data.

The main contribution of this study is the evaluation of geospatial clustering of IBC rates in the contiguous United States, revealing patterns in the spatial incidence of the disease from 2004 to 2012. The study examines the associations with compositional characteristics of the study population: race, ethnicity and age; as well as community-level contextual characteristics: poverty, urbanicity, and a new poverty-urbanicity-race composite variable which helps to highlight more specific types of areas. We expect that rural areas may not yield a greater risk of IBC on average, yet may yield a greater risk of IBC in some situations, as captured by the new composite variable. We expect that higher area poverty is associated with greater risk of IBC, both because it increases the likelihood that the cancer patient is poor and because it indicates lower access to quality healthcare resources such as cancer specialists. Finally, we expect that the population-based data will inform us regarding whether person’s age, race or ethnicity is associated with greater risk of IBC, which we can generalize to the US population of women with breast cancer (BC).

## 2. Materials and Methods

### 2.1. Data Source

IBC data was drawn from the United States Cancer Statistics (USCS) database, available at the National Center for Health Statistics Research Data Center, which is a population-based surveillance system of cancer registries with data representing 98% of the US population [10]. Most states participate in the USCS registry data system, but three do not (Kansas, Maryland, Minnesota), and four states do not allow use of county of residence information (Illinois, Michigan, Missouri, Ohio). We excluded these seven states and an additional state, Virginia, because data were not available until 2007. We also excluded Hawaii and Alaska because of missing contextual data, leaving a total of 40 states included in the analysis. The USCS database with geographic identifiers for county of residence is a restricted-use database, accessible only to researchers with specific approved research projects. All results are reviewed before they can be released from the restricted-entry data center where all analyses are conducted. There are limitations regarding what can be examined and reported from the data, to safeguard the privacy of individuals with cancer. This study is the first to use the entire panel of available data to examine geospatial patterning in a rare disease, and what we report here represents the limits of the current allowed use of these data.

This database includes information on demographics, tumor characteristics, and geographic location at time of diagnosis. We examined breast cancer cases diagnosed from 2004 to 2012 and identified instances of IBC from this data. The sample was restricted to all persons having a first breast cancer diagnosis and excluded records when breast cancer was not the primary cancer. Count data for IBC was identified using the Collaborative Stage (CS) Extensions 710 and 730 [11]. Besides geographic location and case identification, two compositional variables were extracted from the database—race and age. Cases were categorized into race and age groups. Race was represented as proportion of the population that is white or black for the focus of this study. The age groups used were as follows: less than 40, 40–49, 50–64, 65–74, and 75 years and older. The IBC incidence data was then modified to create a county-level file that aggregated the total number of IBC cases within each county by Federal Information Processing Standard (FIPS) code. Crude rates were calculated as the count of IBC cases within the county from 2004 to 2012 over the total population of women age 25–84 in the county, per 100,000. The additional contextual county-level data, to inform the social environment, were obtained from many sources, and the data collection and development was supported by a National Cancer Institute grant (1R01CA126858).

**Figure 1:**
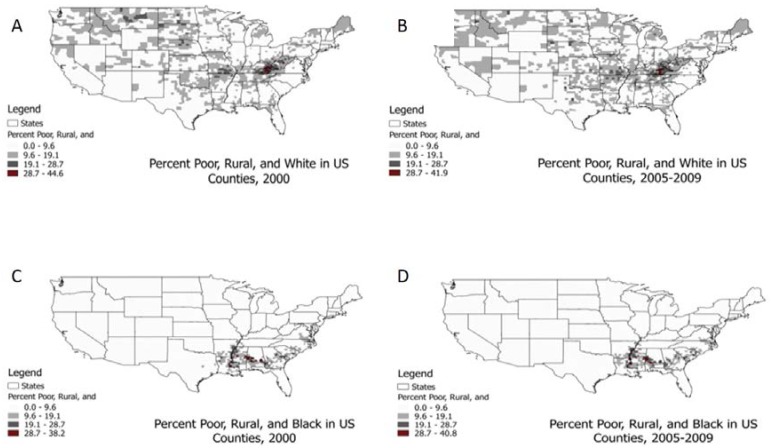
Percent population in counties who are poor, rural, and white (**A**,**B**) or black (**C**,**D**) over time.

Multiple contextual variables were used to explore the role of poverty and urbanicity constructs in the analysis. Unemployed and uninsured rates were the percentage of the area population that is unemployed (percent over age 18) and uninsured (percent under age 65), respectively. Poverty was represented as the percentage of the area population living in poverty, as defined by the US Census based on the Federal Poverty Level. Urbanicity was explored via several variables: (1) proportion of the area population living in a rural area (built up from tracts within counties) and (2) composite variables representing a combination of race, poverty and urbanicity (built using Geographic Information System (GIS) programming from tract-level data within counties). The composite variables represented the percent of the county-population that met three criteria, in socio-economic status (SES), urbanicity, and race/ethnicity dimensions. For example, one of these constructs represents the percent of the county population classified as poor, white, and rural. These composites were created separately for Whites, Blacks, and Hispanics. We analyzed these four composite variables (poor white urban, poor white rural, poor black urban, poor black rural) in addition to the four aforementioned variables. These composite variables represent distinctly different geographic places which are quite persistent over the decade spanning the US Great Recession (Figure 1).

### 2.2. Statistical Analysis

Summary statistics were computed using SAS Software (SAS Institute Inc., Cary, NC, USA) [12]. Descriptive statistics for the IBC study population’s race and age were calculated and included in Table 1. A shapefile was created and spatial analyses were performed in GeoDa software (Open Source) [13,14] and results were mapped in QGIS (Open Source Geospatial Foundation Project) [15]. Following the approach in Mobley et al. and Schieb et al., the global Moran’s I and Local Indicators of Spatial Association (LISA) spatial clustering tests were performed using GeoDa software [16,17]. The Moran’s I test determines if there is global clustering in the pattern of IBC rates; a rejection of the hypothesis of spatial randomness predicates use of the LISA test for the identification of local clusters. There are four types of spatial clusters identified using the LISA statistic: high-high (counties with higher than average rates adjacent to higher than average rates), low-low, high-low, and low-high. Positive spatial autocorrelation in IBC rates among counties is represented by both high-high and low-low clusters, while negative spatial autocorrelation is represented by high-low and low-high clusters [14]. Statistical significance for the LISA statistics is established by bootstrapping. The actual correlation between a place and its neighbors, as defined by a weights matrix, is compared to a thousand or more correlations between the place and sets of randomly chosen neighbors from among all the possible counties. If the actual correlation is far in the tail of the bootstrapped distribution, the actual correlation is determined to be extremely unlikely to have occurred by chance. The hypothesis of spatial randomness is rejected and the county is determined to have statistically significant local spatial autocorrelation with its neighbors.

**Figure 2:**
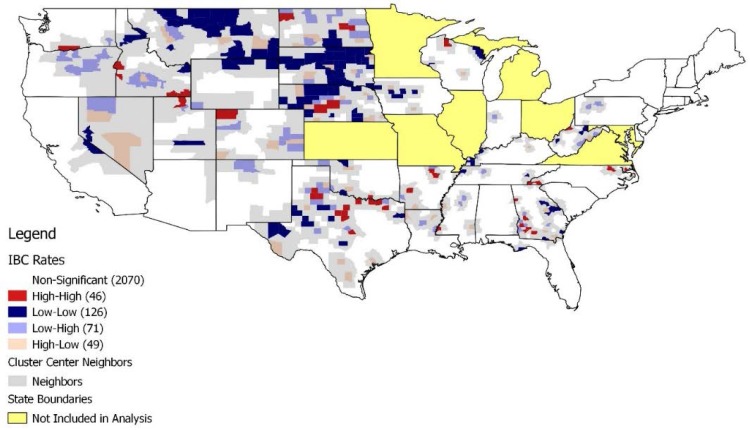
LISA Cluster Map of IBC Rates aggregated from 2004 to 2012.

The results of the LISA analysis were mapped, showing only those counties whose correlations with neighbors were considered to be statistically significant. The LISA map shows the central counties in these significant clusters (e.g., red for high-high), while the actual extent of the cluster includes the central county and its surrounding neighbors as defined by the queen weights matrix (Figure 2). The neighbors are properly included in the cluster, shown here as a grey buffer zone around the center. Two groups were defined for further comparisons: high-rate cluster centers (red) and low-rate cluster centers (blue). Both the compositional and contextual variables of these two areas were then compared. Due to the non-normality of the IBC rate distribution, the more conservative Mann-Whitney U test was employed (rather than the standard *t*-test) to determine whether there were statistically significant differences in the means of the distributions of the variables of interest across the two types of groups/areas (Table 2). Due to the nature of the data, only frequencies, means and standard deviations may be reported to protect the confidentiality of the data.

## 3. Results

### 3.1. Descriptive Statistics

Sample statistics for the cancer cases are provided in Table 1, where a total of 20,388 cases of IBC were identified in the time period studied, and these accounted for 1.25% of all breast cancer cases diagnosed. The mean crude rate of IBC among counties was 28.64 per 100,000 (SD = 28.56; *n* = 2362). Among the examined counties, 57 had rates higher than 100 cases per 100,000 females age 25–84, which demonstrates wide variability of crude IBC rates within the US. Additional demographic variables describing the IBC population are included in Table 1. Approximately 70% of the IBC cases were white, and most cases occurred in the 50–64 age group (41%). However, of all the age groups, those younger than 40 had the highest proportion of IBC cases out of all breast cancer cases, 2.18%, whereas the lowest contribution of IBC, 0.99%, to the total breast cancer cases was in the age group 65–74 (data not shown).

### 3.2. LISA Test Results

Using the Moran’s I test, statistically significant positive global clustering was found (*p* = 0.024), which suggests that the IBC incidence rates were too similar across neighboring counties in some local areas to have occurred by chance. Thus, we used the LISA test to determine where the statistically significant local spatial clusters were located. Figure 2 shows a map of all of the identified positively correlated spatial county clusters using a local significance level of α = 0.05. There were 46 high rate cluster centers (colored red) compared to 126 low rate cluster centers (colored blue). Low rate clusters were apparent in the Midwestern and Northwestern United States. The population in this area is largely homogenous with regards to race, with approximately 85%–90% or higher of the population being white. Additionally, these areas have a much larger American Indian/Alaskan Native population compared to other regions of the United States. The low-rate cluster types were found in 16 of the 40 states. High rate clusters, while few, were apparent in generally rural regions of the United States, and 13 states had this cluster type. Notably, areas near Dallas, Texas, southern Georgia, and North Carolina appeared to carry the bulk of these high-rate clusters. In terms of racial composition, the South carries a much larger proportion of the black population compared to the Northwest and Midwest. Overall, the Northwest and Midwest part of the United States had primarily low-rate clusters, while the South had a mixture of both high and low rate clusters. With the exception of West Virginia, no positively spatially-correlated significant clusters were found in the Northeast.

### 3.3. Comparison of High and Low Cluster Centers

The crude mean rate in the high rate cluster centers was approximately 12-fold greater than the rate in the low rate cluster centers (Table 2). When we compare high and low rate cluster centers, high-rate cluster centers had a larger proportion of black women with IBC as compared to low-rate cluster centers (9% vs. 3%). Age distributions of IBC cases did not differ between high and low-rate cluster centers.

In terms of area context, the high-rate centers had a higher percent of unemployed and higher percent of population in poverty. There is also evidence to suggest that high-rate county clusters had a lower proportion of rural residents compared to its low-rate counterparts. Statistically significant differences were not found with area uninsured rates or the area poor-white-rural composite variable. However, high-rate county clusters had a larger percentage of the area population being poor-black-rural, poor-black-urban, and poor-white-urban.

## 4. Discussion

Our main finding is evidence of geospatial clustering of crude population IBC rates in the examined counties across the United States. The study focused on clusters that were positively spatially autocorrelated, as those that are negatively spatially autocorrelated are considered spatial outliers while the former are considered spatial clusters. A high-high or low-low cluster, in this sense, is more informative as the cluster center and neighbors have higher than or lower than average rates of IBC, respectively. While those diagnosed with IBC were primarily white, the proportion of those diagnosed with IBC that were black in high-rate centers was approximately 3 times that of low-rate cluster centers (0.09 vs. 0.03). This is expected because IBC rates are greater among black women [1,2,5]. The high rate cluster centers had a lower proportion of those who lived in rural areas, whereas earlier published results from Tunisia suggest greater rates in rural areas [18]. Additionally, in the high-rate cluster centers we found a larger percent of the county population that was unemployed and in poverty.

The data were consistent with findings from the literature with IBC rates ranging from 1% to 5% of BC cases, as we found that IBC accounted for approximately 1.25% of all breast cancer cases in the study population. A recent study found that variations in incidence estimates of IBC could be the result of varying diagnosis standards. The authors found that when they used clinical criteria alone IBC accounted for 8.1% of all breast cancer cases while the standard criteria only captured 2.1% of IBC cases. This finding indicates that IBC is likely underestimated in the United States [19]. We used a conservative definition of IBC based only on CS Extension codes as opposed to a broader, more extensive definition which might include diagnostic confirmation codes, histologic type ICD-O-3 codes, behavior codes and recodes for analysis, and perhaps laterality and grade codes. We recognize that the conservative definition used here likely undercounted the number of cases in the time period and study population. Future studies should continue to explore the discrepancy between the diagnostic criteria used to define IBC and variability in what coding practices are being used by the independent state registries. As the study of IBC advances, we expect that coding and diagnostic standards may converge among registries in the US and elsewhere.

While women age 50–64 comprised the bulk of the study population, IBC concentration was found in earlier age groups. Specifically, IBC accounted for approximately 2.18% of all breast cancer cases among women younger than 40 as compared to the overall 1.25%. This finding is expected as earlier studies showed that the age-distribution of IBC cases is shifted toward younger ages [20]. Further geospatial studies should examine the association of IBC diagnosis with age, and how this may vary across geography. Given that younger women are more likely to be diagnosed late-stage due to screening recommendations in the United States and more aggressive types of BC incidence are found among younger age groups (e.g., triple negative and basal-like), it is crucial that we determine risk associated with age, as well as other risk factors [21,22,23,24].

It is important to explore further the link between poverty, urban-rural residence, and IBC diagnosis. We expect to find higher IBC rates in poorer areas. Whether incidence is also higher among more rural populations is of continued interest as studies use more comprehensive definitions of IBC and perhaps catch cases not diagnosed by CS Extension codes alone. We find that high-rate clusters tend to have higher-poverty counties and fewer rural counties than low-rate cluster counties. However, we expect that all rural areas are not the same (Figure 1) and that it may be important to examine poverty by urban-rural and by race. With this refined analysis using our new composite variable, we see that rural aspects may be important for poor black communities while not important for poor white communities. That is, when we examine the composite poor-urbanicity-race variables, high-rate cluster centers were located in counties with a higher percent of those considered poor-black-rural (2.91% vs. 1.28%), poor-black-urban (0.69% vs. 0.11%), and poor-white-urban (1.07% vs. 0.57%) than what was found in low-rate cluster centers, on average. There was no statistically significant difference found in the poor-white-rural population distribution comparison of high- and low-rate centers. Together, these findings may suggest that risk of IBC may be more closely related to poverty than residence along the urban-rural continuum.

It is also worth noting that, although the high-rate IBC clusters have higher percentages of poor-rural-black populations than the low-rate IBC clusters, only some of the hotspots for IBC incidence rates (Figure 2) coincide with the geographic areas of *highest* concentrations of poor rural blacks (Figure 1). Therefore, one cannot conclude from these descriptive analyses that the high-rate clusters are in places with highest concentrations of poor rural blacks (or poor urban blacks, or poor urban whites). One can only conclude that the high-rate and low-rate cluster locations themselves differ significantly in the average percent of poor rural blacks (or poor urban blacks, poor urban whites) in their component counties.

It is also important to note that all the variables related to poverty and rural/urban status are contextual, thus can only describe the area or environment of interest, not the actual characteristics of the study population. Data on actual income status is not available in the USCS database used for this study. Future studies should explore additional variables that represent poverty and urbanicity as compositional variables—i.e., defined by the characteristics of the study population themselves, rather than the neighborhoods they live in—to adequately establish the association between this risk factor and IBC diagnosis. To our knowledge, no population-based cancer registry data exist in the US providing income or SES characteristics of cancer patients. Poverty status may be the most crucial risk factor as it affects many additional factors, such as access to care, screening, transportation, living conditions and housing quality, and employment, which can in turn affect rates of rare BC diagnosis.

Future research may also be concerned with other patient-specific details such as unique biomarkers that would enable patient-centered treatments and allow comparative effectiveness research of various cancer treatments. To this end, a cancer population-based dataset has been developed for cancer populations in eight states and two partial states. The dataset provides detailed treatment and biomarker information from the year 2011 for breast, colon, and rectal cancers as well as chronic myeloid leukemia cases. These cases are collected from ten geographically diverse registries, however no person-level data on income or other SES variables are included [25]. The main limitation of these specialized data is that they do not describe the entire population and would not be useful for analyzing and displaying spatial clusters of rare cancers (or other outcomes), as we have done here. Given ongoing privacy concerns, doing so requires large numbers of cases achieved via aggregation across time and all possible geographic regions. The USCS database that we use here, while limited in several respects, does allow large population-scale studies of geospatial cancer patterns and their underlying contextual characteristics.

## 5. Conclusions

No study in the literature has used such a comprehensive cancer registry database nor these statistical methods paired with geospatial analysis to study this particular outcome. As expected, IBC accounted for a small percentage of all breast cancer cases. There was evidence of geospatial clustering of disease, suggesting that there may be additional environmental risk factors at play, such as air or water or soil contamination by pollutants—which are beyond the scope of this paper. Future studies should continue to explore these environmental factors where possible, and other potential risk factors at the person level (such as poverty, health behaviors, health insurance availability, distance to provider, genetic factors, parity) in order to better capture and potentially intervene in IBC diagnosis. While IBC may be unpreventable, the key is to determine risk factors that may aid in the early capture of the disease, improving survival, due to its difficulty in diagnosis and aggressive nature. Continued use of the United States Cancer Statistics database can provide vital information to fill some of the gaps in knowledge surrounding cancer health disparities, particularly the geospatial patterning of rare cancer types like IBC.

## Figures and Tables

**Table 1:** Descriptive Statistics for the IBC incident cases from 2004 to 2012 (*n* = 20,388).

Variables	IBC Cases, *n* (%)
Age (years)	
<40	1752 (8.59%)
40–49	3956 (19.40%)
50–64	8434 (41.37%)
65–74	3337 (16.37%)
75+	2909 (14.27%)
Race	
White	14,267 (69.98%)
Black	3504 (17.19%)
Hispanic	1966 (9.64%)
Other	651 (3.19%)

**Table 2:** Descriptive Statistics for the Counties included in Study Population: Women Diagnosed with IBC between 2004 and 2012 (*n* = 2362).

Variable	All Counties (*n* = 2362), Mean (SD)	High-Rate Cluster Centers (*n* = 46), Mean (SD)	Low-Rate Cluster Centers (*n* = 126), Mean (SD)	*p* Value ^a^
IBC Rate ^b^	28.64 (28.56)	62.71 (38.97)	5.17 (9.61)	<0.0001
*Study population*				
Race (proportion)				
White	0.84 (0.18)	0.86 (0.16)	0.89 (0.18)	0.034
Black	0.08 (0.14)	0.09 (0.17)	0.03 (0.11)	0.000
Age (years)				
<40	0.04 (0.02)	0.04 (0.03)	0.03 (0.03)	0.146
40–49	0.16 (0.05)	0.13 (0.05)	0.14 (0.09)	0.569
50–64	0.38 (0.07)	0.36 (0.06)	0.36 (0.13)	0.716
65–74	0.23 (0.07)	0.26 (0.08)	0.23 (0.09)	0.050
75+	0.19 (0.06)	0.21 (0.08)	0.23 (0.11)	0.217
*County area contextual variables*				
percent unemployed	5.41 (1.80)	5.00 (1.48)	4.51 (1.84)	0.007
percent uninsured	19.07 (6.23)	21.15 (6.57)	21.25 (6.82)	0.898
percent in poverty	16.12 (6.74)	16.9 (5.16)	15.69 (6.72)	0.048
proportion rural	0.60 (0.31)	0.65 (0.28)	0.77 (0.28)	0.012
percent poor-black-rural	2.42 (5.13)	2.91 (5.7)	1.28 (4.30)	0.000
percent poor-black-urban	0.65 (1.97)	0.69 (2.65)	0.11 (0.58)	0.014
percent poor-white-rural	8.37 (5.57)	9.22 (4.98)	9.29 (4.74)	0.988
percent poor-white-urban	1.32 (2.27)	1.07 (2.03)	0.57 (1.74)	0.018

^a^ *p* values are calculated from the Mann-Whitney U Statistical test comparing high-rate cluster centers to low-rate cluster centers in each covariate; ^b^ count of IBC cases within the county from 2004 to 2012 over the total population of women age 25–84 in the county, per 100,000; SD: standard deviation.
